# Cx32 exerts anti-apoptotic and pro-tumor effects via the epidermal growth factor receptor pathway in hepatocellular carcinoma

**DOI:** 10.1186/s13046-019-1142-y

**Published:** 2019-04-04

**Authors:** Yuke Xiang, Qin Wang, Yunquan Guo, Hui Ge, Yile Fu, Xiyan Wang, Liang Tao

**Affiliations:** 1 Department of Pharmacology, Zhongshan School of Medicine, Sun Yat-Sen University, Guangzhou, 510080 People’s Republic of China; 2 Tumor Research Institute, Xinjiang Medical University Affiliated Tumor Hospital and State Key Laboratory, Urumqi, 830000 People’s Republic of China

**Keywords:** Connexin32, Hepatocellular carcinoma, Apoptosis, EGFR, Src, Nonjunctional function

## Abstract

**Background:**

Abnormal expression or distribution of connexin 32 (Cx32) is associated with hepatocarcinogenesis, but the role of Cx32 and the underlying mechanisms are still unclear.

**Methods:**

The expression level of Cx32 in 96 hepatocellular carcinoma (HCC) specimens was determined using western blotting and immunohistochemistry. The correlation between Cx32 expression and clinicopathological parameters was analyzed. The cell apoptosis rate was examined using flow cytometry and western blotting. The role of Cx32 in the Src kinase and epidermal growth factor receptor (EGFR) signaling pathways was measured by quantitative real-time PCR, western blotting and coimmunoprecipitation (CO-IP). The effect of Cx32 overexpression on the streptonigrin (SN)-induced tumor growth suppression and apoptosis was assessed in nude mice.

**Results:**

Our study showed that overexpressed Cx32 accumulated in the cytoplasm and that Cx32-containing gap junctions (GJs) were nearly absent in HCC specimens. Upregulated Cx32 expression was highly correlated with advanced tumor-node-metastasis (TNM) stage and poor tumor differentiation and was an independent predictive marker for poor prognosis in HCC. Overexpression of Cx32 significantly inhibited SN-induced apoptosis by activating the EGFR signaling pathway in vitro and in vivo. Moreover, the expression levels of Cx32 and EGFR were positively correlated in HCC specimens. The CO-IP experiments demonstrated that Cx32 could bind to Src kinase, and the western blotting results revealed that Cx32 increased the levels of EGFR and p-EGFR by upregulating Src expression.

**Conclusion:**

The present study demonstrated that overexpressed and internalized Cx32 was associated with advanced TNM stage and poor tumor differentiation and predicted poor prognosis in HCC. Cx32 facilitated HCC progression by blocking chemotherapy-induced apoptosis in vitro *and* in vivo via interacting with Src and thus promoting the phosphorylation of EGFR, subsequently activating the EGFR signaling pathway. Cx32 may be a potential biomarker and a new therapeutic target for HCC.

**Electronic supplementary material:**

The online version of this article (10.1186/s13046-019-1142-y) contains supplementary material, which is available to authorized users.

## Background

Connexins (Cxs) normally assemble into gap junction (GJ) channels on the surfaces of two adjacent cells [1] and participate in maintaining homeostasis by mediating the direct intercellular exchange of molecule [2]. However, during carcinogenesis, GJs generally reduced in number or lost due to decreased expression levels and/or internalization of Cxs [3]. In some cancers, restoration of GJ by increasing cytomembrane Cx expression suppresses tumor development [4] and sensitizes cells to radiotherapy/chemotherapy [5]. Thus, Cx-containing GJs are believed to suppress tumor progression [6, 7]. Recently, emerging evidence has demonstrated that Cxs per se might play a proper nonchannel role in the development of carcinoma [8] by altering the expression of different genes [9, 10]. However, the nonjunctional role of Cxs in carcinogenesis remains unclear.

Although the diagnostic criteria and clinical therapeutic strategies have evolved in recent decades, hepatocellular carcinoma (HCC) remains the second most common cause of cancer-related death worldwide [11, 12]. Therefore, identifying a novel predictive biomarker and effective target is imperative for improving HCC therapy. Normal hepatocytes mainly express Cx32, which accounts for approximately 90% of the total connexin population and forms GJ channels in the human liver [13]. GJs have been verified to be generally absent in HCC, and the recovery of Cx32-containing GJs results in sensitization to suicide gene therapy [14]. However, the conclusion about the alterations and role of Cx32 in hepatocarcinogenesis remains controversial. Some literature has shown that Cx32 is a liver tumor suppressor. For example, studies in Cx32-deficient mice demonstrated that Cx32 deletion promotes hepatocarcinogenesis [15, 16]. Besides, overexpression of Cx32 was reported to suppresse the metastatic and proliferative protential of hepatoma cells [17]. However, Cx32 has also been reported to be liver tumor promoter. Cytoplasmic accumulation of Cx32 promotes the invasion and metastasis of HCC cells [18] and expands the cancer stem cell population in HuH7 cells by enhancing self-renewal [19]. A few reports have verified that Cx32 expression is involved in the promotion of tumors by phenobarbital [20, 21]. In summary, some of these documents lack clinicopathological data, and some do not distinguish the nonchannel function of Cx32 from the function of Cx32-containing GJs in vitro. Thus, the question of whether Cx32 contributes to hepatocarcinogenesis remains to be resolved.

Here, we found that Cx32, which generally forms GJs on the surface of hepatocytes, was abnormally upregulated and internalized in HCC tissues and was closely associated with poor prognosis. Upregulated Cx32 suppressed chemotherapy-induced apoptosis by interacting with Src kinase and activating the epidermal growth factor receptor (EGFR) signaling pathway independent of GJs in vitro. These findings suggest that Cx32 proteins play a crucial role in the progression of HCC.

## Methods

### Reagents

Anti-Cx32, Cx43 and Cx26 antibodies for immunohistochemistry were purchased from BOSTER (Wuhan, China). PV-9000 DAB detection Kit was obtained from ZSGB-Bio (Beijing, China). The DEME (high glucose) and RPMI-1640 media, Lipofectamine™ 3000, anti-Cx26 antibody were purchased from Invitrogen (Carlsbad, CA, USA). 2-aminoethoxydiphenyl-borate (2-APB), streptonigrin (SN), polybrene, anti-Cx43, anti-β-tubulin antibodies, and HRP-conjugated secondary antibodies were acquired form Sigma-Aldrich (St. Louis, MO, USA). Polyethylenimine (PEI) was purchased from Polysciences, Inc. (Warrington, PA, USA). Annexin V-FITC / PI apoptosis detection kit was from Biotool (Houston, TX, USA). TRIzol was obtained from Life Technologies (California, USA). cDNA Synthesis SuperMix kit and qPCR SuperMix kit were purchased from Transgen Biotech (Beijing, China). BCA protein assay kit was from Bio-Rad (Hercules, CA, USA). Chemiluminescent HRP Substrate Kit was from Millipore Corporation (Billerica, MA, USA). Anti-Cx32 antibody was purchased from Santa Cruz (Dallas, TX, USA). Anti-GAPDH antibody was obtained from Ray Antibody Biotech (Beijing, China). EGFR, p-EGFR (Tyr845), PARP, cleaved-Caspase3, Erk 1/2, p-Erk 1/2 (Thr202/Tyr204), STAT3, p-STAT3 (Tyr705), Bcl-2, Bak, Bax, and Src primary antibodies were obtained from Cell Signaling Technology (Danvers, MA, USA). Nonspecific mouse or rabbit IgG was purchased from Beytime (Shanghai, China). Protein G Plus/Protein A Agarose Suspension was from Merck Millipore (Billerica, MA, USA). HRP-conjugated secondary antibodies (light chain specific or heavy chain specific) were obtained from Abbikine (Wuhan, China).

### Tissue specimens and clinical data

From May 2011 to November 2013, 96 HCC patients underwent hepatectomy at the Affiliated Cancer Hospital of Xinjiang Medical University, Xinjiang, China. All the flesh HCC specimens, corresponding peritumoral tissue (< 3 cm distance from the tumor tissue), and remote normal liver tissues (5 cm away from the tumor tissue) were collected within 10 min after hepatectomy and then stored in liquid nitrogen for protein extraction and paraffin embedding. The cohort of 13 female and 83 male patients had a median age of 53.53 ± 10.88 years (range 24 ~ 78 years), with a median follow-up time of 27.5 months (range 2~ 44 months). The mean tumor size was 7.50 ± 3.53 cm (range: 1.5~20 cm). All patients and their corresponding tissue samples had been confirmed by pathology. None of the patients had received any chemoradiotherapeutic agents in the preoperation. Clinical variables including age, sex, pathological differentiation, TNM stages, serum alpha-fetoprotein (AFP), presence of hepatitis B surface antigen (HBsAg), and tumor size were recorded. Histological grading, according to the Edmondson-Steiner (ES) criteria, showed that ES grade I (well-differentiated), II (moderately differentiated), III (poorly differentiated) and IV (undifferentiated) tumors were found in 21 (21.8%), 36 (37.5%), 24 (25.0%), and 15 (15.6%) cases. Cancer clinical staging was performed according to the AJCC/UICC tumor–node–metastasis (TNM) stage (2010), which showed that TNM stage I, II, and III tumors were found in 15 (15.6%), 18 (18.7%), and 63 (65.6%) cases.

### Immunohistochemistry (IHC)

IHC was performed as described previously [22]. Sections were incubated overnight at 4 °C with primary antibody (1:100) and were then visualized using a PV-9000 DAB detection kit according to the manufacturer’s protocol. Sections were counterstained with hematoxylin and observed under a BX-51 microscope (Olympus, Tokyo, Japan). The degree of immunostaining in the sections was reviewed and scored independently by 2 observers based on both the percentage of positive-stained tumor cells and the staining intensity. The number of positive cells was divided into five grades (percentage scores): ≤10% (0), 11–25% (1), 26–50% (2), 51–75% (3), and > 75% (4). The staining intensity was graded in four categories on a scale from 0 to 3 (intensity scores): no staining (0), light-brown staining (1), brown staining (2) and dark-brown staining (3). Protein staining was evaluated using the following formula: overall staining score = intensity score × percentage score. A final score of ≤4 was defined as negative staining, and a final score of > 5 was defined as positive staining.

### Cell culture and high−/low-density cultures

HCC cells were purchased from the American Type Culture Collection (Manassas, VA, USA). HepG2 cells and SMMC-7721 cells were cultured in DMEM (high-glucose) and RPMI-1640 medium, respectively, supplemented with 10% fetal bovine serum (FBS), 100 U/ml penicillin and 100 U/ml streptomycin at 37 °C in an atmosphere containing 5% CO_2_. For low-density cultures, 1 × 10^5^ cells were seeded in a 150 mm dish to physically inhibit GJ formation (the cells were not in direct contact with each other). For high-density cultures, 1 × 10^5^ cells seeded in each well of a 6-well plate were allowed to form GJs [22, 23]. In high-density cultures, 2 h of pretreatment with 50 μm 2-APB was used to inhibit the function of GJs (Additional file 1: Figure S2) [22–24].

### Apoptosis induction and analysis

Apoptosis was induced in cells by stimulation with 1 μM streptonigrin (SN) for 7 h [22, 23]. Overall apoptosis was measured by staining cells using an Annexin V-FITC / PI apoptosis detection kit according to the manufacturer’s protocol, followed by analysis using a FACScan (Beckman Instruments, Fullerton, CA, USA). Early apoptotic cells were considered to be Annexin V-positive but PI-negative. Cell apoptosis was analyzed using FlowJo 7.6 software.

### RNA interference

Cells were seeded and grown to 30–50% confluence, and complexes of sequence-nonspecific siRNAs (NC) or targeted siRNAs (50 nM) (RiboBio, Guangzhou, China) and 5 μl of Lipofectamine™ 3000 were added to cells in each well according to the manufacturer’s instructions. Cells were incubated for an additional 48 h for the experiments. The sequences of the synthesized Cx32, EGFR and Src siRNA were as follows, and siCx32_2, siEGFR_1, siSrc_1 were chosen for subsequent experiments.

siCx32_1: 5′-CCGGCATTCTACTGCCATT-3′, siCx32_2: 5′-GGCTCACCAGCAACACATA-3′, siCx32_3: 5′-GCAACAGCGTTTGCTATGA-3′. siEGFR_1: 5′-GGCTGGTTATGTCCTCATT-3′, siEGFR_2: 5′-CCTTAGCAGTCTTATCTAA-3′, siEGFR_3: 5′-GGAACTGGATATTCTGAAA-3′. siSrc_1: 5′-CAAGAGCAAGCCCAAGGAT-3′, siSrc_2: 5′-CAGGCTGAGGAGTGGTATT-3′, siSrc_3: 5′-GCAGTTGTATGCTGTGGTT-3′.

### Plasmid transfection and establishment of stable cell lines

For transient transfection, cells were seeded and grown to 80% confluence. Following the manufacturer’s instructions, 2.5 μg of each DNA plasmid was mixed with 5 μl of Lipofectamine™ 3000 and 5 μl of P3000™. Then, the mixture was added to the cell culture medium. Cells were incubated for an additional 48 h for the experiments. Plasmid vectors overexpressing Cx32 (EX-A0514-M02–5), EGFR (EX-A0275-M98–5), or Src (EX-B0107-M09) along with the corresponding control vectors, were constructed by GeneCopoeia (Rockville, MD, USA).

To generate stably transfected cells, SMMC-7721 cells were transfected with lentiviral plasmids containing Cx32 (EX-A0514-Lv105) or with empty vector (pEZ-Lv105), which were constructed by GeneCopoeia, and HepG2 cells were transfected with lentiviral plasmids containing the shCx32 sequence or with the negative control vector (pLVX-shRNA-tdTomato-Puro), which were constructed by Landbiology (Guangzhou, China). Lentiviral particles were produced by transfecting 293 T cells with 6 μg of lentiviral plasmid, 4.5 μg of psPAX2 and 1.5 μg of pMD2.G using 30 μl of PEI (100 μM) in a 10 cm culture plate. Then, HCC cells were incubated in medium containing the virus and polybrene (6 μg/ml) for 48 h. After infection, cells were selected by culture with puromycin (2 μg/ml) for 2 weeks.

The sequences for the short-hairpin RNA targeting Cx32 (shCx32) was as follows: 5′-GCTGCAACAGCGTTTGCTACTCGAGTAGCAAACGCTGTTGCAGCTTTTTTT-3′.

### Quantitative real-time polymerase chain reaction (qPCR)

After total RNA extraction using TRIzol, RNA concentrations were measuring using a Nanodrop 2000 (Thermo Fisher, Waltham, MA, USA). Reverse transcription to cDNA was performed using a cDNA Synthesis SuperMix kit on a C1000 Thermal Cycler (Bio-Rad, CA, USA). Next, cDNA samples (2 μl) were used for qPCR with the qPCR SuperMix kit, and amplification was conducted for 40 cycles on a StepOnePlus Real-Time PCR System (Applied Biosystems, Foster City, CA, USA). The cycle time (Ct) values of the selected genes were first normalized to the CT value for GAPDH for the same sample, and fold changes were calculated by relative quantification (2^-ΔΔCt^). The following formula was used: 2^-ΔΔCt^ = 2^control group (Ct value of target gene - Ct value of GAPDH) - experiment group (Ct value of target gene - Ct value of GAPDH)^. All experiments were repeated 3 times. The primer sequences synthesized by Sangon Biotech Co., Ltd. (Shanghai, China) were as follows:

EGFR: 5′-CCCACTCATGCTCTACAACCC-3′ (Forward), 5′-TCGCACTTCTTACACTTGCGG-3′ (Reverse); Src: 5′-TGGCAAGATCACCAGACGG-3′ (Forward), 5′-GGCACCTTTCGTGGTCTCAC-3′ (Reverse); Cx32: 5′-ACACCTTGCTCAGTGGCGTGA-3′ (Forward), 5′-AGGGACCACAGCCGCACATGG-3′ (Reverse); GAPDH: 5′-TGTGGGCATCAATGGATTTGG-3′ (Forward), 5′-ACACCATGTATTCCGGGTCAAT-3′ (Reverse).

### Western blotting (WB)

Cells were washed with cold PBS and then harvested in cell lysis buffer. Whole cell lysates were sonicated and were then centrifuged at 12,000 rpm for 30 min at 4 °C. The protein concentration was determined using a BCA protein assay kit. Fifteen micrograms of protein from each sample was separated by SDS-PAGE followed by transfer to a nitrocellulose membrane. Membranes were blocked with 5% (*w*/*v*) skim milk and were then incubated with primary antibodies (1:1000) overnight at 4 °C. Immunoreactive bands were visualized using a Chemiluminescent HRP Substrate Kit and scanned with an ImageQuant LAS 4000™ (GE Healthcare, Piscataway, NJ, USA). The expression values for each target protein were normalized to the corresponding GAPDH or β-tubulin expression values.

### Coimmunoprecipitation (CO-IP)

Total protein was extracted in cell lysis buffer. The supernatant was incubated with 2 μg of anti-Cx32, anti-p-EGFR or anti-Src antibody on a spinning wheel at 4 °C for 4 h, respectively, with parallel samples containing 2 μg of nonspecific mouse or rabbit IgG as the negative controls (IgG group). Additionally, a certain proportion of supernatant was incubated without any antibody as the positive control (input group). Next, 30 μl of Protein G Plus/Protein A Agarose Suspension was added dropwise to bind to the antibodies overnight at 4 °C. The beads were washed 5 times with lysis buffer and resuspended in sample buffer, followed by boiling for 5 min. The samples were separated by SDS-PAGE and reacted with the corresponding primary antibodies (1:1000), followed by incubation with HRP-conjugated (light chain specific or heavy chain specific) secondary antibodies (1:5000).

### Xenograft tumor model

BALB/c-nu mice (male, 4 weeks of age, 18–20 g) were purchased from Hunan SJA Laboratory Animal Co., Ltd. (Hunan, China). All experimental procedures were approved by the Institutional Animal Care and Use Committee of Sun Yat-sen University. The nude mice were first randomly divided into 2 groups (*n* = 12). These two groups were subcutaneously inoculated with stable cell lines, including SMMC-Vector cells and SMMC-Cx32 cells (5 × 10^6^ in 200 μl of PBS), near the right scapula. When the tumors had grown to an appropriate size (200 mm^3^), each group was further randomized into 2 groups (*n* = 6) according to the tumor volume and body weight. The control group was orally administered vehicle (PBS), and the treatment group was orally administered 0.5 mg/kg SN (dissolved in PBS) once every two days for a total of 7 treatment [25]. Tumors sizes were as measured daily using a caliper, and body weights were recorded. Tumor volumes were calculated using the following formula: V = (A × B^2^)/2, where V is the volume (mm^3^), A is the long diameter, and B is the short diameter (both in mm). Mice were sacrificed 24 h after the final intragastric administration.

### Immunofluorescence, Cytomembrane protein extraction and Parachute dye-coupling assay

The details are shown in Additional file 3 (Supplemental Methods).

### Statistical analysis

All experiments were repeated at least three times. Parametric data were analyzed by one-way ANOVA or Student’s t-test, and nonparametric data were analyzed by Fisher’s exact test or the Chi-square test. The association between Cx32 expression and clinicopathological characteristics was statistically determined using the Pearson χ^2^ test. The Kaplan-Meier method and log-rank test were applied to generate and compare survival curves. A Cox proportional hazards model was used to determine independent factors for survival based on the variables selected in the univariate and multivariate analyses. A two-tailed value of *P* < 0.05 was considered statistically significant. Quantitative data are presented as the means ± SDs (standard deviations). The data were statistically analyzed by using the SPSS software package (version 16.0).

## Results

### Cx32 is overexpressed and internalized in HCC tissues

**Fig. 1:**
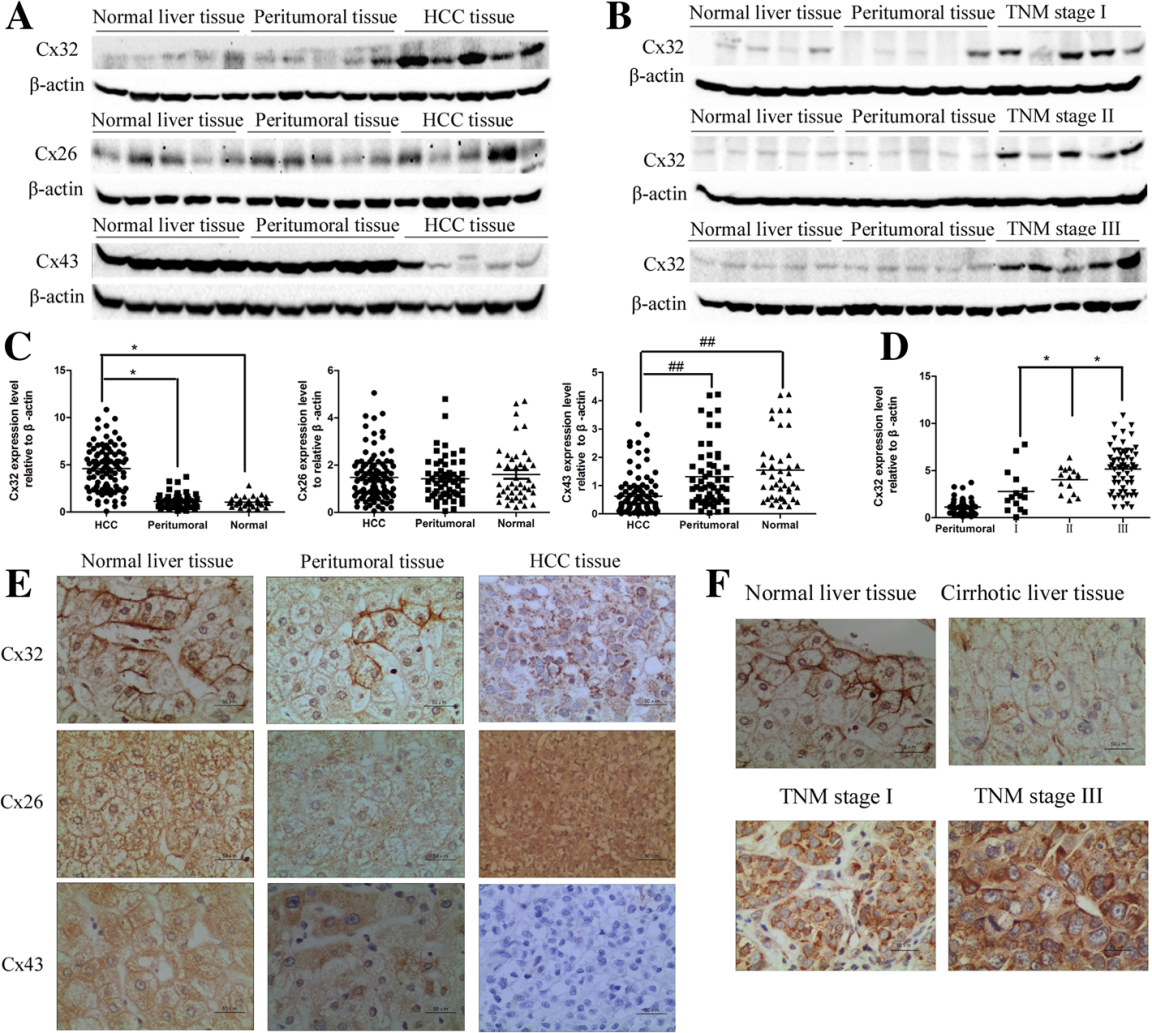
Expression and distribution of Cx32, Cx26 and Cx43 in patients with HCC. **a.** The protein expression levels of Cx32, Cx26 and Cx43 were determined by western blot analysis. β-actin was used as the loading control. **b.** The expression of Cx32 was correlated with increased TNM stages, as revealed by western blot analysis. β-actin was used as the loading control. **c**. Statistical analysis of the relative expression levels of Cxs in HCC tissues, peritumoral tissues, and normal liver tissues. ^**^, *P* < 0.01; ^##^, *P* < 0.01. **d.** Statistical analysis of the relative expression levels of Cx32 in peritumoral tissues and HCC tissues with different TNM stages. ^*^, *P* < 0.05. **e.** Representative IHC staining of Cx32, Cx26 and Cx43 protein in normal liver tissues (left panels), peritumoral tissues (middle panels) and HCC tissues (right panels) (400×). Scale bars: 50 μm. **f.** Representative IHC staining of Cx32 in normal liver tissues, cirrhotic tissues and early and advanced HCC tissues (400×). Scale bars: 50 μm

Initially, we evaluated the expression of three major hepatocellular Cx isoforms— Cx32, Cx43 and Cx26—in specimens of human HCC tissues (*n* = 96), peritumoral tissues (*n* = 57) and remote normal liver tissues (*n* = 43) by western blotting. As shown in Fig. 1 a and c, in HCC tissues, Cx32 expression significantly increased compared with that in peritumoral tissues (*P* < 0.01) and normal liver tissues (*P* < 0.01). In HCC tissues, the expression of Cx26 was unchanged (*P* > 0.05), whereas the expression of Cx43 was significantly decreased (*P* < 0.01). Notably, Cx32 expression increased with increasing tumor-node-metastasis (TNM) stage (stage I versus stage II, *P* < 0.05; stage II versus stage III, *P* < 0.05; stage I versus stage III, *P* < 0.01) (Fig. 1 b and d).

Next, the expression levels (Additional file 2: Table S1) and localization (Fig. 1 e) of these Cxs were examined by IHC. Cx32 was prominently localized in cytomembranes in normal liver tissues (40/42, 95.2%) and peritumoral tissues (85/96, 88.5%), and dense distributions were observed in the junctions of adjacent cells, which were identified as Cx32-constituted GJ plaques. However, in HCC tissues, Cx32 was significantly distributed in the cytoplasm (80/96, 83.3%), implying the loss of Cx32-containing GJ in HCC. Cx26 was mainly distributed in cytomembranes in normal and peritumoral tissues but was evenly localized in both cytomembranes and the cytoplasm in HCC tissues. Cx43 was uniformly located in cytomembranes and the cytoplasm in normal tissues and peritumoral tissues but its levels were greatly decreased in HCC tissues, as previously reported [26, 27]. We further explored the alteration in the Cx32 distribution during the progression of HCC development. As shown in Fig. 1 f, Cx32 was mainly localized on the cell membrane in normal liver tissues and cirrhotic liver tissues but was constantly translocated to the cytoplasm in early HCC (TNM stage I) and advanced HCC (TNM stage III) tissues. In summary, upregulated Cx32 was translocated to the cytoplasm and was associated with advanced TNM stages. However, Cx26 was diffusely distributed in cytomembranes and the cytoplasm, and Cx43 was almost nonexistent in HCC tissues. The mislocalization or downregulated expression of these Cxs implied that GJ function was seriously impaired in HCC.

### High Cx32 expression predicts a poor prognosis in HCC

**Fig. 2:**
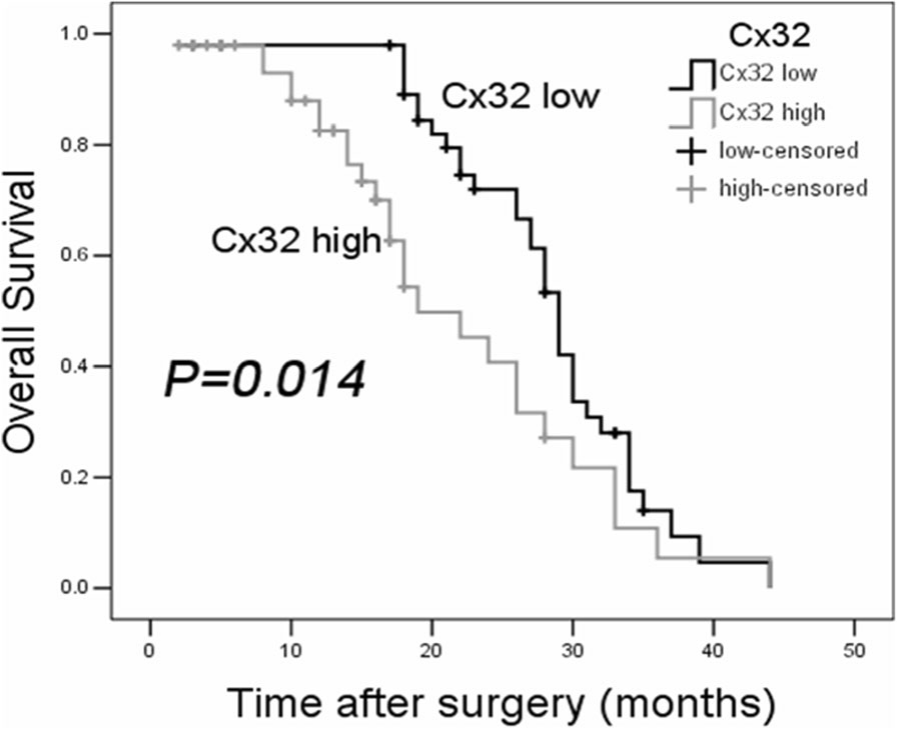
Kaplan-Meier analysis. Patients in the low Cx32 group (*n* = 48) had significantly longer overall survival (OS) times than those in the high Cx32 group (*n* = 48) (*P* = 0.014, log-rank test)

We focused on whether Cx32 overexpression was correlated with poor HCC prognosis. Based on the mean Cx32 protein expression level (relative mean expression level of Cx32 = 4.5952 ± 0.2371) tested by western blotting in 96 HCC specimens, the data were classified into the Cx32-low expression group (*n* = 48) and Cx32-high expression group (*n* = 48). The Pearson χ^2^ test showed that Cx32 expression was strongly correlated with clinical TNM stage (*r* = 0.373, *P* < 0.05) and tumor differentiation (*r* = 0.488, *P* < 0.05) (Table 1). Furthermore, Kaplan-Meier survival analysis revealed that patients with high Cx32 expression had shorter overall survival (OS) than patients with low Cx32 expression (*P* = 0.014) (Fig. 2). Moreover, univariate analysis showed that poor differentiation (*P* = 0.042), positive HBsAg status (*P* = 0.034) and high Cx32 expression (*P* = 0.001) were significantly correlated with poor OS (Additional file 2: Table S2), and multivariate analysis indicated that high Cx32 expression (*P* = 0.001) was an independent predictor of poor prognosis in HCC patients (Additional file 2: Table S3). Therefore, cytoplasmic overexpressed Cx32 was closely associated with the malignant progression of HCC and was an independent predictor of poor prognosis.

**Table 1:** Relationship between Cx32 expression and clinicopathologic variables in 96 HCC tissues

Clinicopathologic parameters	n	Cx32 expression		*χ* ^*2*^	*P*	r
		Low	High			
All cases	96	48	48			
Age (years)						
≤ 50	40	19 (39.6%)	21 (43.8%)			
>50	56	29 (60.4%)	27 (56.3%)	0.171	0.679	–
Sex						
male	83	41 (85.4%)	42 (87.5%)			
female	13	7 (14.6%)	6 (12.5%)	0.089	0.765	–
Serum AFP (ng/ml)						
≤ 25	36	17 (35.4%)	19 (39.6%)			
>25	60	31 (64.6%)	29 (60.4%)	0.178	0.673	–
Tumor size (cm)						
≤ 5	26	14 (22.9%)	12 (25%)			
>5	70	34 (70.8%)	36 (75%)	0.211	0.646	–
HBsAg						
Negative	15	9 (19.6%)	6 (12%)			
Positive	81	37 (80.4%)	44 (88%)	1.040	0.308	–
Vascular invasion						
absent	89	45 (93.8%)	44 (91.7%)			
present	7	3 (6.3%)	4 (8.3%)	0.154	0.695	–
Differentiation						
Well-moderate (1–2 grade)	57	40 (80.3%)	17 (35.4%)			
Poor-differentiate (3–4 grade)	39	8 (16.7%)	3 (64.6%)	22.854	0.000	0.488
TNM Stage						
I + II	33	25 (52.1%)	8 (16.7%)			
III	63	23 (47.9%)	40 (83.3%)	13.345	0.000	0.373
Liver Cirrhosis						
absent	66	30 (62.5%)	36 (75%)			
present	30	18 (37.5%)	12 (25%)	1.745	0.186	–

### Cx32 affects the expression of Bcl-2 family proteins

**Fig. 3:**
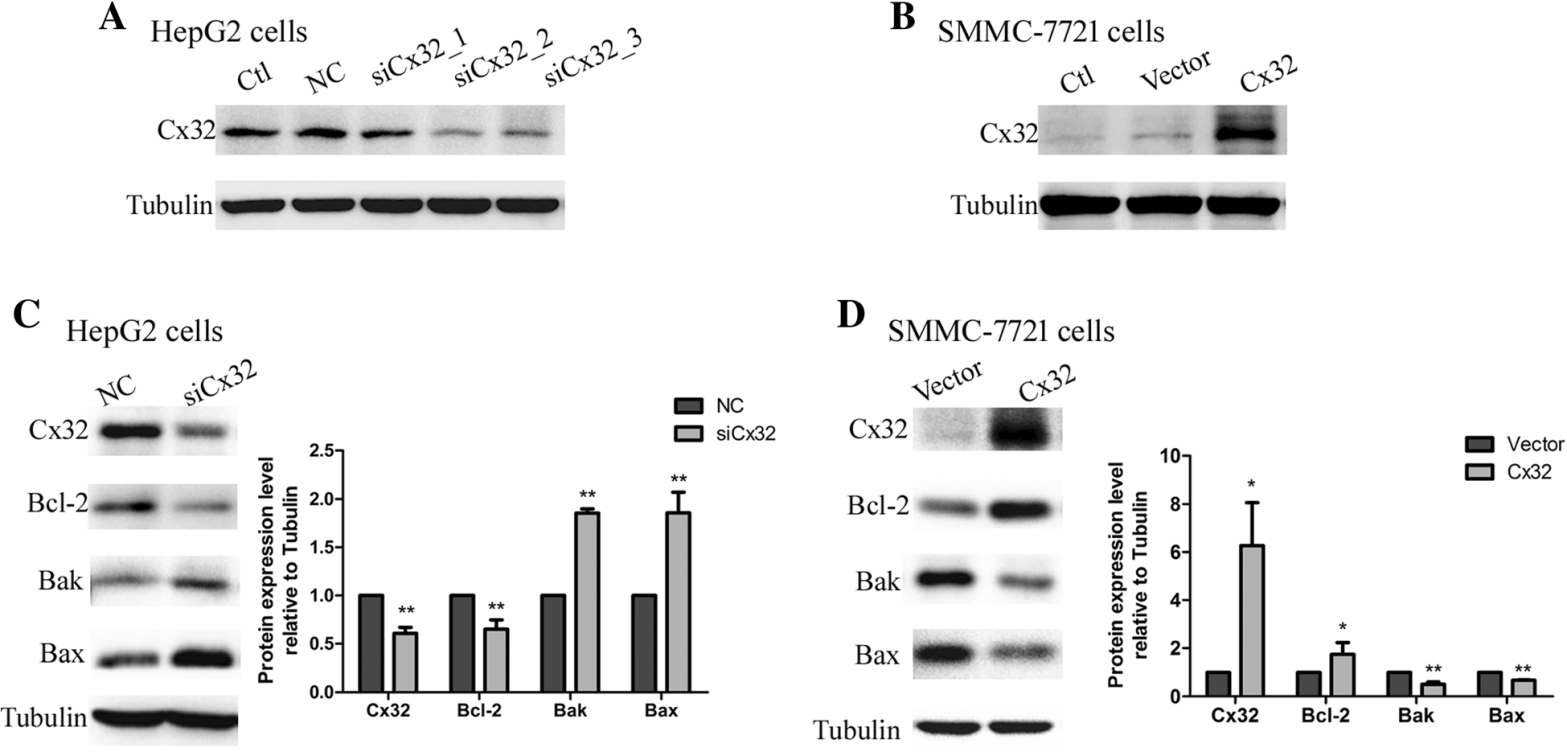
Cx32 regulates the expression of Bcl-2 family proteins in HCC cell lines. **a.** Cx32 expression was knocked down in HepG2 cells by siRNA transfection. siCx32_2 showed the greatest efficiency in reducing Cx32 expression. **b.** Transient plasmid transfection into SMMC-7721 cells induced Cx32 overexpression. **c.** Silencing Cx32 expression in HepG2 cells increased the expression levels of Bax and Bak but decreased the expression level of Bcl-2 (*n* = 3). ^**^, *P* < 0.01 versus NC. **d.** Overexpression of Cx32 in SMMC-7721 cells caused the upregulation of Bcl-2 expression and the downregulation of Bax and Bak expression (n = 3). ^**^, *P* < 0.01 versus Vector

To elucidate the involvement of upregulated and mislocalized Cx32 in HCC progression, two HCC cell lines were used—HepG2 cells, with a high expression level of Cx32 and functional GJs, and SMMC-7721 cells, with a lower expression level of Cx32 and barely functional GJ (Additional file 1: Figures S1 and S2). Cx32 expression in HepG2 cells was knocked down by transfection with Cx32-siRNA (siCx32_1–3); siCx32_2 showed the greatest efficiency in silencing Cx32 expression (Fig. 3 a and Additional file 1: Figure S3). Moreover, Cx32 expression was upregulated in SMMC-7721 cells by transfection with Cx32-expressing vectors (Fig. 3 b). The immunofluorescence results showed that transfection of the Cx32-expressing vector mainly enhanced the cytoplasmic overexpression of Cx32 (Additional file 1: Figure S3). Silencing the expression of Cx32 reduced the expression of the anti-apoptotic protein Bcl-2 but enhanced the expression of pro-apoptotic proteins such as Bax and Bak in HepG2 cells (Fig. 3 c). The opposite effects of overexpression of Cx32 on the above apoptosis-related proteins were observed in SMMC-7721 cells (Fig. 3 d). Therefore, we further focused on whether the accumulated cytoplasmic Cx32 played a role in the apoptosis of HCC cells in a GJ-independent manner.

### Cx32 protects HCC cells from apoptosis in a GJ-independent manner

**Fig. 4:**
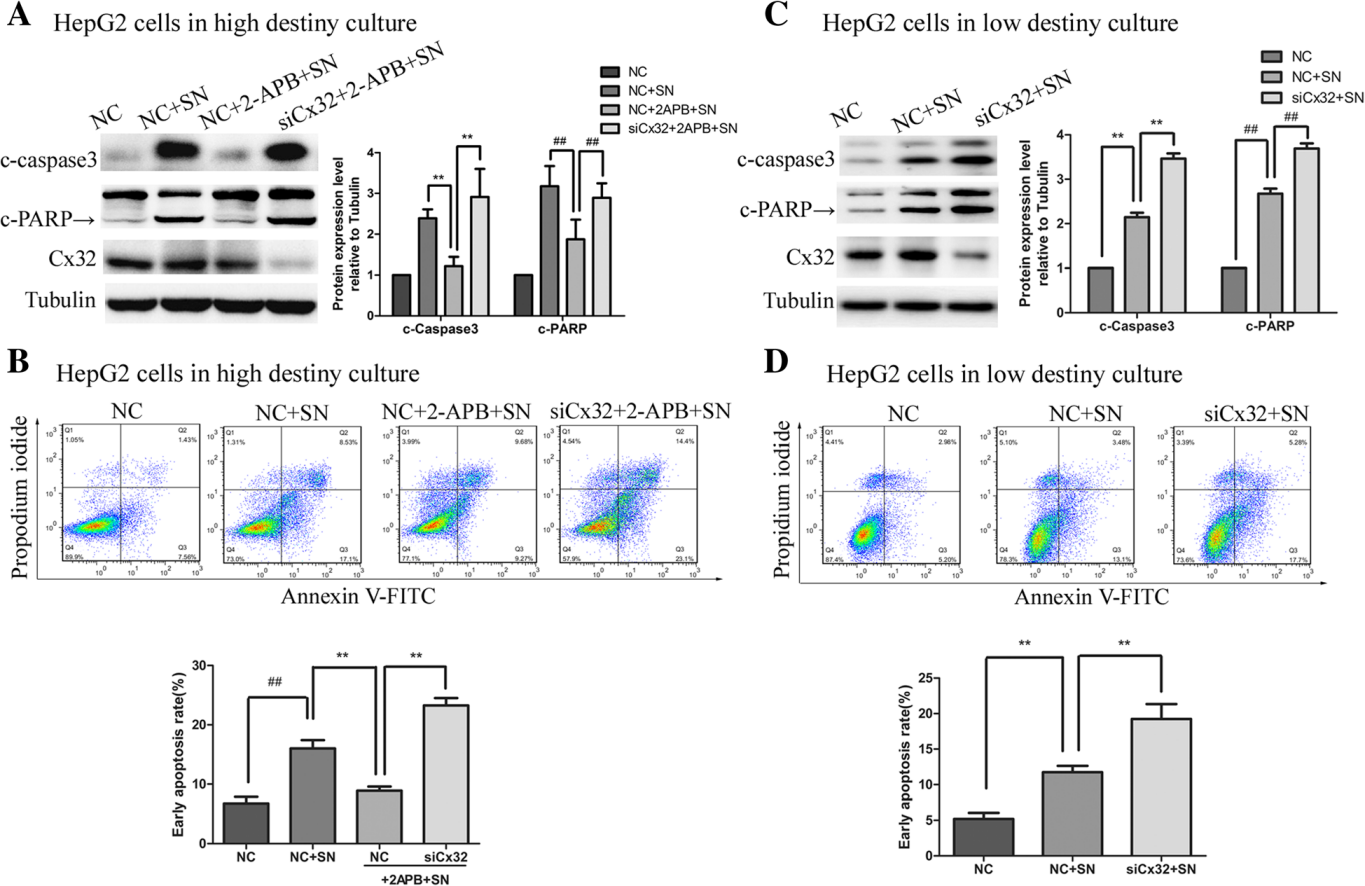
Cx32 exerts an anti-apoptotic effect on HepG2 cells in a GJ-independent manner. **a.** When GJ function was inhibited by pretreatment with 2-APB (50 μm, 2 h), knockdown of Cx32 promoted the SN-induced increase in the levels of cleaved-caspase3 and cleaved-PRAR in HepG2 cells, as revealed by western blot analysis (n = 3). ^**^, *P* < 0.01; ^##^, *P* < 0.01. **b.** Silencing the expression of Cx32 promoted SN-induced apoptosis in HepG2 cells when GJ function was inhibited by 2-APB, as assessed by flow cytometry (n = 3). ^##^, *P* < 0.01 versus NC; ^**^, *P* < 0.01 versus NC + 2-APB + SN. **c.** When GJs were physically eliminated by low-density culture conditions, Cx32 silencing enhanced the SN-induced increase in the levels of cleaved-caspase3 and cleaved-PARP in HepG2 cells, as revealed by western blot analysis (n = 3). ^**^, *P* < 0.01; ^##^, *P* < 0.01. **d.** Cx32 silencing facilitated SN-induced apoptosis in HepG2 cells in low-density culture, as assessed by flow cytometry (n = 3). ^**^, *P* < 0.01 versus NC + SN

The early apoptosis rate of cells was examined by flow cytometry, and the levels of cleaved-PARP and cleaved-caspase 3, two well-known protein markers of apoptosis, were assessed by western blotting. As shown in Fig. 4 a and b, noticeable apoptosis of HepG2 cells was induced by SN (1 μM, 7 h) but was significantly suppressed by pretreatment with the GJ inhibitor 2-APB (50 μM, 2 h) (NC + SN versus NC + 2-APB + SN, *P* < 0.01). Furthermore, knockdown of Cx32 in HepG2 cells markedly reversed the anti-apoptotic effect of 2-APB (siCx32 + 2-APB + SN versus NC + 2-APB + SN, *P* < 0.01). These results implied that under conditions of suppressed GJ function, Cx32 expression antagonized SN-induced apoptosis. Similarly, in HepG2 cells grown in low-density culture conditions (with no GJ formation), Cx32 silencing noticeably exacerbated SN-induced apoptosis (siCx32 + SN versus NC + SN, *P* < 0.01) (Fig. 4 c and d). These results indicate that cytoplasmic Cx32 exerts an anti-apoptotic effect in HepG2 cells, in a GJ-independent manner.

**Fig. 5:**
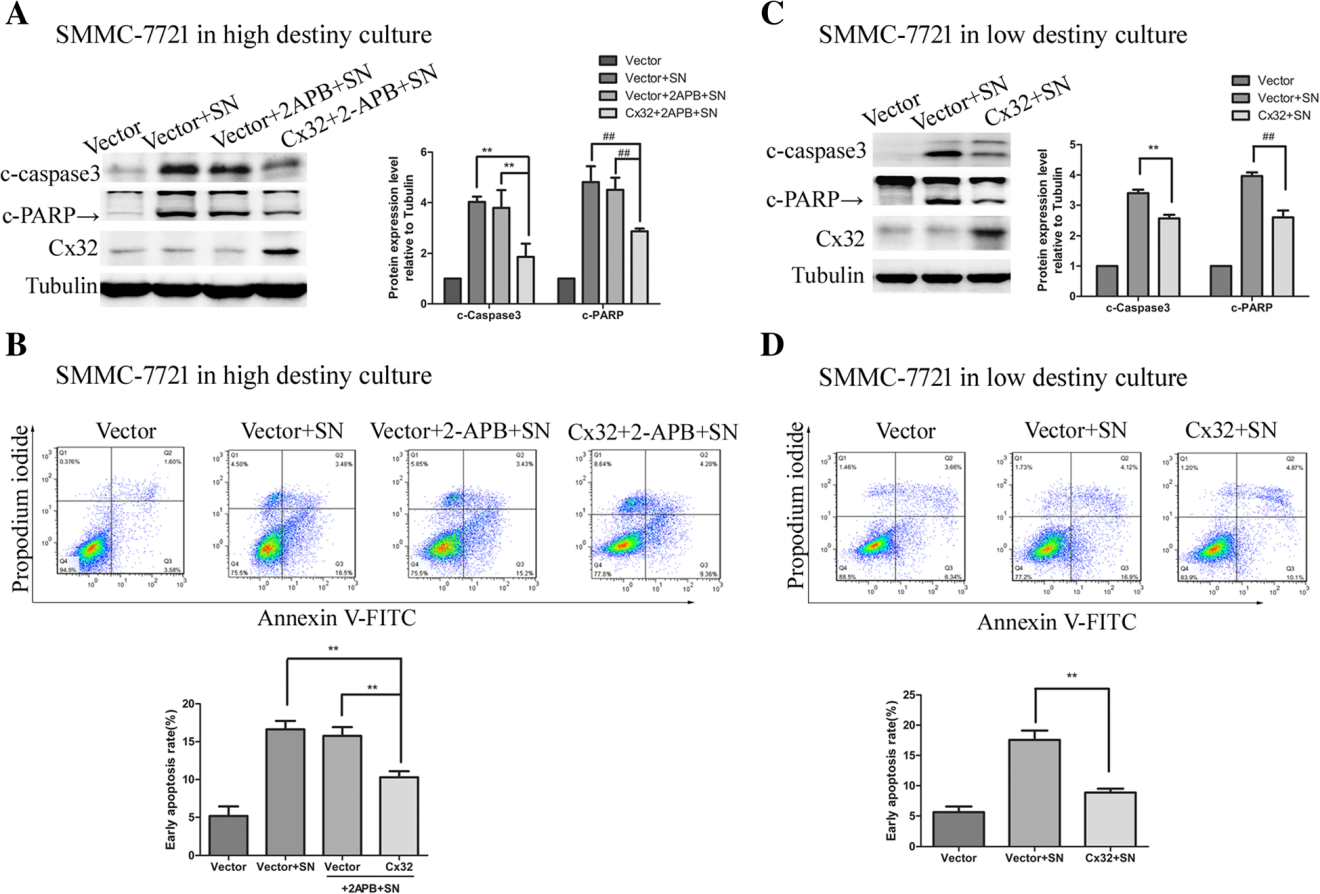
Overexpressed Cx32 exerts an anti-apoptotic effect on SMMC-7721 cells in a GJ-independent manner. **a.** When GJ function was inhibited by pretreatment with 2-APB, overexpression of Cx32 alleviated the SN-induced increase in the levels of cleaved-caspase3 and cleaved-PARP in SMMC-7721 cells, as revealed by western blot analysis (n = 3). ^**^, *P* < 0.01; ^##^, *P* < 0.01. **b.** Upregulation of Cx32 expression suppressed SN-induced apoptosis in SMMC-7721 cells when GJ function was inhibited by 2-APB, as revealed by flow cytometry (*n* = 3). ^**^, *P* < 0.01 versus Cx32 + 2-APB + SN. **c.** When GJ were physically eliminated by low-density culture conditions, Cx32 overexpression reduced SN-induced increase in the levels of cleaved-caspase3 and cleaved-PARP in SMMC-7721 cells (*n* = 3). ^**^, *P* < 0.01; ^##^, *P* < 0.01. **d.** Upregulation of Cx32 expression inhibited SN-induced apoptosis in SMMC-7721 cells in low-density culture, as revealed by flow cytometry (*n* = 3). ^**^, *P* < 0.01 versus Cx32 + SN

As shown in Fig. 5 a and b, due to the low expression of Cx32 and weak GJ function in SMMC-7721 cells (Additional file 1: Figure S1 and S2), 2-APB pretreatment barely reversed the SN-induced apoptosis (Vector+SN versus Vector+ 2-APB + SN, *P* > 0.05), but Cx32 overexpression in SMMC-7721 cells significantly attenuated SN-induced apoptosis (Cx32 + 2-APB + SN versus Vector+ 2-APB + SN, *P* < 0.01). More convincingly, Cx32 overexpression significantly suppressed apoptosis (Cx32 + SN versus Vector+SN, *P* < 0.01) in SMMC-7721 cells grown at a low cell density (with no GJ formation) (Fig. 5 c and d). In summary, under circumstances of impaired GJ function, Cx32 proteins exerts an intrinsic anti-apoptotic effect in HCC cells.

### Cx32 exerts an anti-apoptotic effect via the EGFR signaling pathway

**Fig. 6:**
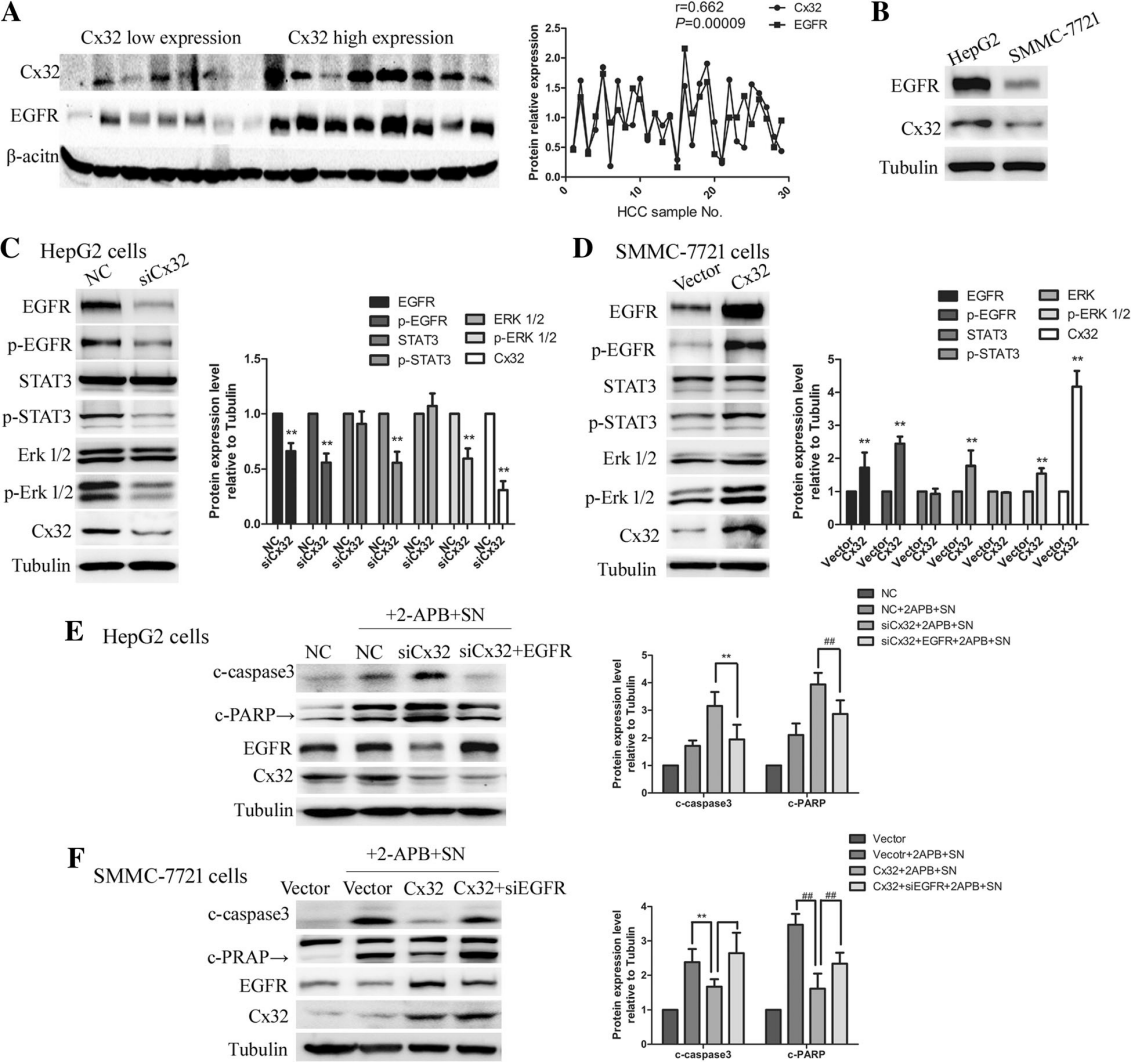
Cx32 exerts anti-apoptotic effects by activating EGFR signaling pathway. **a.** In 30 HCC specimens, the expression of EGFR was significantly correlated with the expression of Cx32 (*r* = 0.662, *P* < 0.01)**. b.** The expression level of EGFR was significantly higher in HepG2 cells than in SMMC-7721 cells. **c** and **d.** The effects of Cx32 knockdown or overexpression on the EGFR signaling pathway in HepG2 cells and SMMC-7721 cells were determined by western blot analysis (*n* = 3, respectively). ^**^, *P* < 0.01 versus HepG2 NC (**c**) or SMMC-7721 Vector (**d**). **e**. In rescue experiments, cotransfection of siRNA-Cx32 and EGFR-expression vectors into HepG2 cells reversed the pro-apoptotic effects of Cx32 knockdown. ^**^, *P* < 0.01; ^##^, *P* < 0.01. **f.** In rescue experiments, cotransfection of Cx32 expression vectors and siRNA-EGFR into SMMC-7721 cells reversed the anti-apoptotic effects of Cx32 overexpression. ^**^, *P* < 0.01; ^##^, *P* < 0.01

The EGFR signaling pathway plays a vital role in cell proliferation, apoptosis and cell survival. To characterize the mechanisms by which Cx32 protects HCC cells from apoptosis, we investigated the possible involvement of the EGFR signaling pathway. EGFR expression was positively correlated with Cx32 expression (*r* = 0.662, *P* < 0.01) in 30 human HCC specimens (Fig. 6 a). Similarly, in HCC cell lines, EGFR protein expression in high-Cx32-expression HepG2 cells was much higher than that in low-Cx32-expression SMMC-7721 cells (Fig. 6 b). Furthermore, downregulation of Cx32 by siCx32_2 caused a significant decrease in the levels of EGFR, p-EGFR, p-STAT3 and p-Erk 1/2 in HepG2 cells but did not change the expression levels of total STAT3 and Erk 1/2 (Fig. 6 c). Overexpression of Cx32 in SMMC-7721 cells led to the opposite effect; the levels of EGFR, p-EGFR, p-STAT3 and p-Erk 1/2 were enhanced but those of total STAT3 and Erk 1/2 were unchanged (Fig. 6 d). In summary, the expression levels of Cx32 and EGFR were positively correlated in HCC specimens and cell lines, and overexpressed Cx32 contributed to the activation of the EGFR signaling pathway.

Next, rescue experiments were performed to verify whether EGFR was crucial for the Cx32-mediated anti-apoptotic effect in HCC cell lines. As shown in Fig. 6 e, the pro-apoptosis phenotype induced by transfection of siCx32_2 was restored by cotransfection of siCx32_2 and EGFR-overexpressing vectors in HepG2 cells. Moreover, the anti-apoptotic effect caused by Cx32 overexpression was reversed by cotransfection of siEGFR and Cx32-overexpressing vectors in SMMC-7721 cells (Fig. 6 f). Taken together, these data suggest that Cx32 exerts its anti-apoptotic effect via the EGFR signaling pathway.

### Interaction between Cx32 and Src contributes to the EGFR activation

**Fig. 7:**
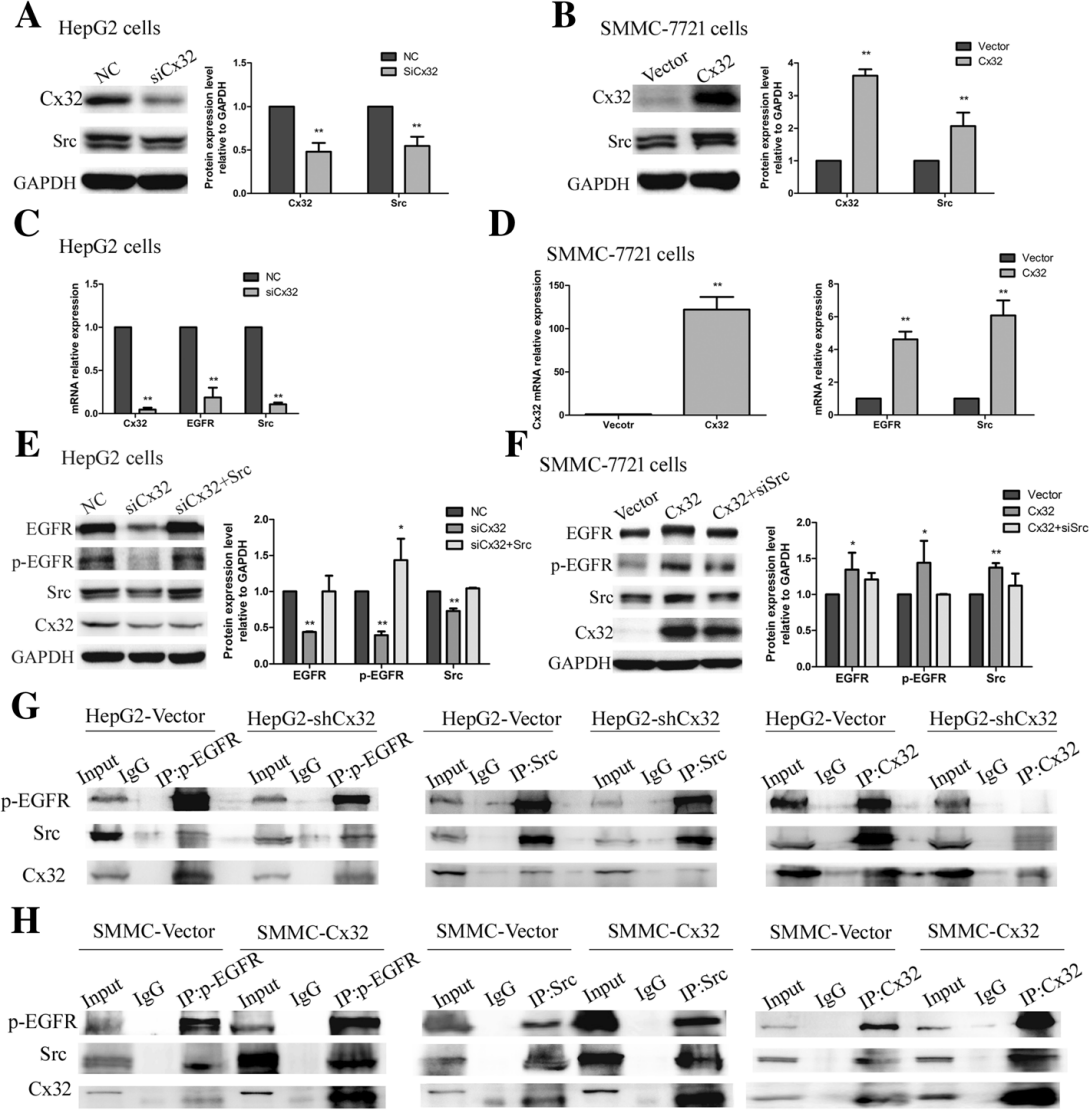
Cx32 upregulates the expression and activation of EGFR by binding with Src. **a** and **b.** In HCC cell lines, silencing or overexpressing Cx32 caused Src downregulation or upregulation, respectively. ^**^, *P* < 0.01 versus HepG2 NC (**a**) or SMMC-7721 Vector (**b**). **c**. Silencing Cx32 by siCx32 transfection caused a decrease in the EGFR and Src mRNA levels in HepG2 cells, as determined by qPCR. GAPDH was used as the loading control. ^**^, *P* < 0.01 versus NC. **d**. Overexpression of Cx32 in SMMC-7721 cells upregulated the mRNA levels of Cx32 (left panel), EGFR and Src (right panel), as determined by qPCR. GAPDH was used as the loading control. ^**^, *P* < 0.01 versus Vector. **e**. The decrease in the levels of EGFR, p-EGFR and Src mediated by siCx32 was reversed by cotransfection of the Src overexpression vector in HepG2 cells. ^**^, *P* < 0.01 versus NC. **f.** The increase in the levels of EGFR, p-EGFR and Src induced by Cx32 overexpression was rescued by cotransfection of siSrc in SMMC-7721 cells. ^**^, *P* < 0.01 versus Vector. **g** and **h.** Cx32, p-EGFR and Src interacted with each other, as detected by CO-IP experiments in HepG2 cells (**g**) and SMMC-7721 cells (**h**). Lysate supernatants incubated without antibody, termed Input, were used as the positive control, and proteins precipitated by IgG were used as the negative control

Src kinase has been reported to interact with activated EGFR to form a complex that increases EGFR tyrosine phosphorylation and accelerates cell transformation and cancer development [28]. We further identified the synergistic expression of Cx32 and Src in HCC cell lines (Fig. 7 a and b). Altering the Cx32 mRNA levels by transfection of the siCx32_2 or Cx32 overexpression vector simultaneously affected the mRNA levels of EGFR and Src in HCC cells (Fig. 7 c and d). Subsequently, we performed rescue experiments to investigate whether the upregulation and activation of EGFR mediated by Cx32 depended on Src kinase. As shown in Fig. 7 e, silencing the expression of Cx32 in HepG2 cells decreased the levels of EGFR, p-EGFR and Src, and this effect was reversed by cotransfection with a Src overexpression vector. In addition, the increases in the levels of EGFR, Src and p-EGFR induced by Cx32 overexpression were rescued by cotransfection of siSrc_1 in SMMC-7721 cells (Fig. 7 f). Thus, Cx32 upregulated and activated EGFR by enhancing the expression of Src.

CO-IP experiments were performed to investigate the potential interactions among Cx32, p-EGFR and Src. As shown in Fig. 7 g, significant binding between Cx32, p-EGFR and Src was observed in HepG2-Vector cells, but this binding was weakened in HepG2-shCx32 cells. In contrast, the interaction between Cx32, p-EGFR and Src was intensified in SMMC-Cx32 cells compared with that in SMMC-Vector cells (Fig. 7 h). These findings revealed that Cx32 might bind to p-EGFR and Src to form a complex and then activate the downstream signaling pathway to aggravate tumor malignancy by, for example, preventing cell apoptosis.

### Cx32 alleviates SN-mediated growth inhibition and apoptosis in vivo

**Fig. 8:**
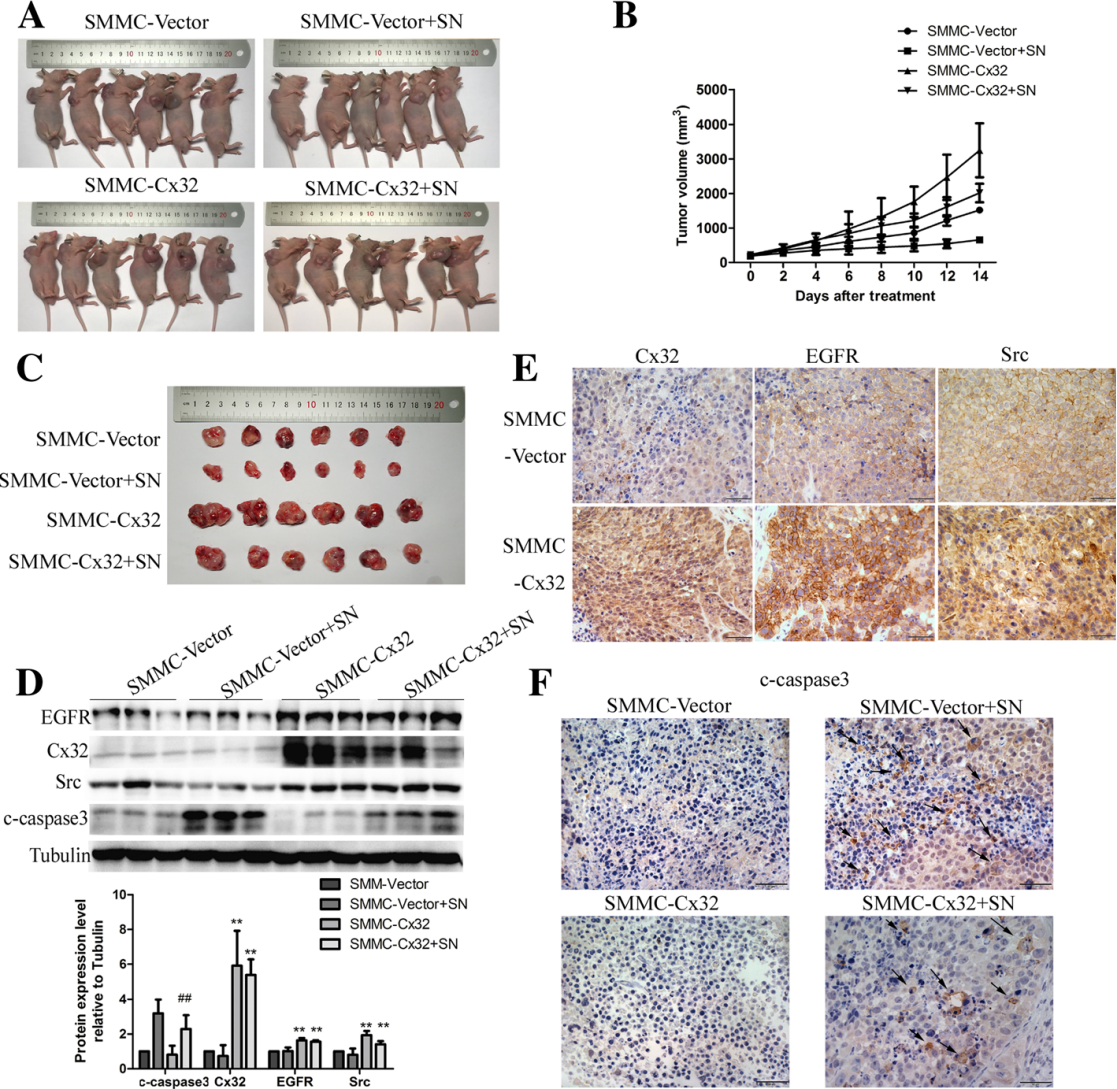
Overexpression of Cx32 promotes the proliferation of SMMC-7721 cells and protects cells from SN-induced apoptosis in vivo. **a.** Representative images of the nude mouse xenograft model. **b.** Tumor growth curves. Overexpression of Cx32 promoted the tumor growth in nude mice and significantly reduced the growth suppression mediated by intragastric injection of SN (0.5 mg/kg). **c.** Representative images of tumors from the sacrificed nude mice. **d.** Representative images of IHC for Cx32, EGFR and Src in tumors generated from SMMC-Vector and SMMC-Cx32 cells. Scale bars: 50 μm. **e.** Overexpression of Cx32 inhibited the SN-induced increase in the levels of cleaved-caspase3 by increasing the levels of EGFR and Src. ^##^, *P* < 0.01 vs Vector +SN. ^**^, *P* < 0.01 versus Vector. **f.** Representative images of IHC for cleaved-caspase3 in each group. Overexpression of Cx32 inhibited the SN-induced increase in the level of cleaved-caspase3. Scale bars: 50 μm

Given the above in vitro results that Cx32 overexpression protects HCC cells from SN-induced apoptosis, we next verified the anti-apoptotic function of Cx32 in an HCC xenograft model. Stable SMMC-Cx32 cells and the corresponding SMMC-Vector cells were subcutaneously injected into nude mice (*n* = 12, respectively). The tumor were allowed to grow to an appropriate size (200 mm^3^), and each group was then further randomized to two groups: the control group (PBS) and the SN-treated group (0.5 mg/kg). As shown in Fig. 8 a and b, the volume of tumors generated from SMMC-Vector cells was appreciably smaller than that of tumors generated from SMMC-Cx32 cells, indicating that overexpression of Cx32 exacerbated progressive growth in vivo. Moreover, the growth inhibition rate achieved by SN treatment in tumors generated from SMMC-Cx32 cells was significantly lower than that in tumors generated from SMMC-Vector cells (Fig. 8 c). Then we assessed the levels of Cx32, EGFR, Src and cleaved-caspase 3 in the tumors by western blotting and IHC. The expression levels of Cx32, EGFR and Src in SMMC-Cx32 tumors were significantly higher than those in SMMC-Vector tumors (Fig. 8 d and e). Moreover, an evident increase in the level of cleaved-caspase 3 was seen in the SMMC-Vector+SN group compared with that in the SMMC-Cx32 + SN group (Fig. 8 d and f). In summary, synergistic expression of Cx32, EGFR and Src was seen in the HCC xenograft model, and overexpression of Cx32 showed significant anti-apoptotic effects in vivo.

## Discussion

Although Cx32-containing GJs were well recognized as a tumor-inhibiting factor, the specific nonjunctional effect of Cx32 on the progression of HCC has rarely been investigated. In this study, we revealed the nonjunctional biological role of Cx32 and identified its specific mechanisms in HCC. Cx32 expression was abnormally increased, and Cx32 was translocated to the cytoplasm from the cytomembrane in human HCC specimens. Upregulated and internalized Cx32 was closely correlated with advanced TNM stage, poor differentiation and predicted poor prognosis in HCC patients. Furthermore, the data revealed a potential molecular mechanism by which Cx32, in a GJ-independent manner, exerts anti-apoptotic effects to protect HCC cells from chemotherapeutic exposure by interacting with Src kinase and activating the EGFR signaling pathway in vitro and in vivo. These findings indicate that internalized and accumulated Cx32 may be a carcinogenic factor and reveal a molecular mechanism underlying the development and progression of HCC.

GJ channels are abundant at cell-cell contact sites in normal tissues, but they generally disappear during carcinogenesis due to the loss or abnormal subcellular localization of Cxs. While the role of Cx-containing GJ channels in suppressing tumorigenesis and enhancing the cytotoxicity of chemoradiotherapy has been thoroughly established [29], the nonjunctional role of Cxs in mediating tumor cell survival or death remains controversial [30–32]. Recently, we reported that overexpressed Cx32 exerts anti-apoptotic effects in human cervical carcinoma cell lines in vitro [22, 23], and Cx32 was subsequently reported to be constantly internalized and accumulated and to play a role in inhibiting cisplatin-induced apoptosis during the establishment of a cisplatin-resistant A2780 ovarian cancer cell line [33]. Our previous findings indicate that Cx32 may be responsible for cancer cell escape from apoptosis.

In a recent study, based on the novel finding that overexpressed Cx32 is internalized and associated with clinicopathological parameters and poor prognosis in HCC tissues, we speculated that cytoplasmic Cx32 may accelerate the occurrence and development of HCC, in a manner depended on the proper function of Cx32 proteins rather than on Cx32-mediated GJ channels. Furthermore, overexpression of Cx32 enhanced the expression level of the anti-apoptotic protein Bcl-2 but reduced the expression levels of the pro-apoptotic proteins Bak and Bax in vitro, suggesting that Cx32 might participate in controlling cell apoptosis. To mimic the clinicopathological characteristics of the absence of GJs in HCC, we inhibited the function or eliminated the formation of GJs in vitro using pharmacological and physical methods (see the Methods section). Cx32 acted as a functional protein that protected HCC cell lines from SN-induced apoptosis after excluding the function of GJ in vitro. Thus, cytoplasmic Cx32 may act as a tumor promoter although cytomembrane Cx32-consistited GJ servers as a tumor suppresser. Our results presents that enhancing the cytomembrane expression but reducing the cytoplasmic expression of Cx32, rather than simply increasing the total expression of Cx32 in HCC cells, might effectively inhibit the progression of HCC. We hypothesize that Cx32 proteins primarily assemble to form GJ channels to maintain homeostasis and suppress tumorigenesis in the normal liver. However, during the progression of HCC, Cx32 proteins internalize and chiefly regulate multiple signaling molecules and pathways, such as Bcl-2 protein family members and the EGFR signaling pathway, to promote tumor survival and prevent apoptosis. The critical discrepancy may involve the dysregulation of progressive Cx proteins trafficking [34], including Cx protein synthesis, cytomembrane trafficking, endocytosis, ubiquitination and degradation [35, 36]. In fact, cytoplasmic mislocalization of Cx proteins has been found in other tumors and may be associated with malignancy [37]. For instance, Marc Mensnil et al. reported that Cx43 was aberrantly expressed in the cytoplasm and that Cx30 was localized in nuclei in a rat glioma cell line, from whence these Cxs played roles in cell growth regulation [38]. Hirofumi Yamamoto et al. showed that cytoplasmic expression of Cx26 was responsible for lung metastasis in colorectal cancer [39]. In future studies, unraveling the role of alteration in Cx trafficking in tumor occurrence and development may be crucial to understand the correlation between Cxs and tumor progression.

Accumulating evidence suggests that high expression and constitutive activation of EGFR are found in most cancers, including HCC [40]. EGFR overactivation may result in the resistance of tumor cells to chemotherapy and radiation treatment, via the activation of the PI3K/Akt, JAK/STAT3 and Ras/Raf/MEK/Erk downstream signaling pathways [41]. Our data showed that in HCC specimens and cell lines, the expression of Cx32 was positively correlated with the expression of EGFR. Overexpression of Cx32 in vitro increased the expression and activation of EGFR and its downstream proteins and vice versa. Moreover, EGFR overexpression rescued the pro-apoptotic effect of Cx32 knockdown, whereas EGFR downregulation reversed the anti-apoptotic effect of Cx32 overexpression. Thus, Cx32 was confirmed to act as an upstream activator of the EGFR pathway and to play key roles in mediating chemotherapy resistance in HCC.

Finally, we investigated the molecular mechanism by which Cx32 regulates the EGFR signaling pathway. The carboxyl-terminal (CT) domain of Cxs contains multiple sites for protein-protein interactions. For instance, the CT domain of Cx43 contains binding sites for the Src homology 3 (SH3) and SH2 domains of Src kinase [42]. Moreover, Cx32 has been reported to interact with the SH3/Hook domain of Discs Large homology 1 (Dlgh1), a PDZ domain-containing scaffolding protein, and to then control cell proliferation [43]. Src kinase is significantly associated with malignant transformation and oncogenesis [44] and mediates EGFR phosphorylation [45], including the phosphorylation of tyrosine residues Y845 [46], Y920 [47] and Y1101 [48]. We found that Cx32 positively regulated the expression of Src and EGFR in HCC cells at transcriptional and translational levels. Subsequently, CO-IP experiments confirmed the protein-protein interactions among Cx32, p-EGFR and Src. In addition, we further verified the correlation among Cx32, EGFR and Src in an HCC xenograft model and confirmed the anti-apoptotic effect of overexpressed Cx32 in vivo. These findings indicate that Cx32 interacts with Src kinase, and subsequently activating the EGFR signaling pathway to protect HCC cells from chemotherapy-induced apoptosis.

## Conclusion

In conclusion, cytoplasmic Cx32 exerted nonjunctional effects to protect HCC cells from chemotherapy-induced apoptosis via binding to Src and activating the EGFR signaling pathway in vitro and in vivo. The elevated expression levels and cytoplasmic mislocalization of Cx32 in HCC indicate that Cx32 may be a biomarker for clinical prognosis and chemotherapy resistance. Identifying an approach to restore the abnormal localization of Cxs is thus important for oncotherapy and deserves further study.

## Additional files

Additional file 1: **Figure S1.** The expression and distribution of Cx32 in HCC cell lines. **Figure S2.** The GJ function in HCC cell lines. **Figure S3.** The expression and distribution of Cx32 in HepG2-NC and HepG2-siCx32, SMMC-7721-vecotr and SMMC-7721-Cx32 cells. (ZIP 4027 kb)

Additional file 2: **Table S1.** Cx32, Cx26 and Cx43 expression detected by immunohistochemical analysis in remote normal liver, peritumoral and HCC tissues. **Table S2.** Univariate Cox proportional hazard analysis for overall survival Variables. **Table S3**. Multivariate Analysis of Different Prognostic Parameters in Patients with HCC by Cox Regression Analysis. (DOCX 17 kb)

Additional file 3: Supplemental Methods: immunofluorescence, cytomembrane protein extraction, parachute dye-coupling assay. (DOCX 14 kb)

## Abbreviations

CO-IP: Co-Immunoprecipitation
Cx: Connexin
EGFR: Epidermal growth factor receptor
GJ: Gap junction
HCC: Hepatocellular carcinoma
IHC: Immunohistochemistry
p-EGFR: phospho-EGFR
