# Supplementary material for: Cx32 exerts anti-apoptotic and pro-tumor effects via the epidermal growth factor receptor pathway in hepatocellular carcinoma
Source: J Exp Clin Cancer Res. 2019 Apr 4;38:145. doi: 10.1186/s13046-019-1142-y (PMC6449973; doi:10.1186/s13046-019-1142-y)
Supplement: Supplementary file 1 — Figure S1. The expression and distribution of Cx32 in HCC cell lines. Figure S2. The GJ function in HCC cell lines. Figure S3. The expression and distribution of Cx32 in HepG2-NC and HepG2-siCx32, SMMC-7721-vecotr and SMMC-7721-Cx32 cells. (ZIP 4027 kb) [file 13046_2019_1142_MOESM1_ESM.zip › Additonal files (Supplementary Figure Legends).docx]

**Supplementary Figure S1. The expression and distribution of Cx32 in HCC cell lines. A.** The total expression of Cx32 in HCC cells was detected by western blot analysis. **B.** The expression of cytomembrane Cx32 and cytoplasmic Cx32 in HCC cells was analyzed by western blot analysis. Na^+^-K^+^-ATPase was used as the cytomembrane control, and tubulin was used as the cytoplasmic control.

**Supplementary Figure S2. The GJ function in HCC cell lines.** The GJ function of HCC cells was assessed by the Parachute dye-coupling assay, and GJ function of HCC cells was significantly inhibited by 2-APB (50 μM, 2 h). The scale bars represent 50 μm.

**Supplementary Figure S3. The expression and distribution of Cx32 in HepG2-NC and HepG2-siCx32, SMMC-7721-vecotr and SMMC-7721-Cx32 cells.** Immunofluorescence of Cx32 protein in HCC cell lines after transfection siCx32 or Cx32-vector is shown (400×). Hoechst33258 was used to stain the nuclei, and phalloidin was used to stain the cytoskeleton.
