# Supplementary figures and images for: Cx32 exerts anti-apoptotic and pro-tumor effects via the epidermal growth factor receptor pathway in hepatocellular carcinoma
Source: J Exp Clin Cancer Res. 2019 Apr 4;38:145. doi: 10.1186/s13046-019-1142-y (PMC6449973; doi:10.1186/s13046-019-1142-y)

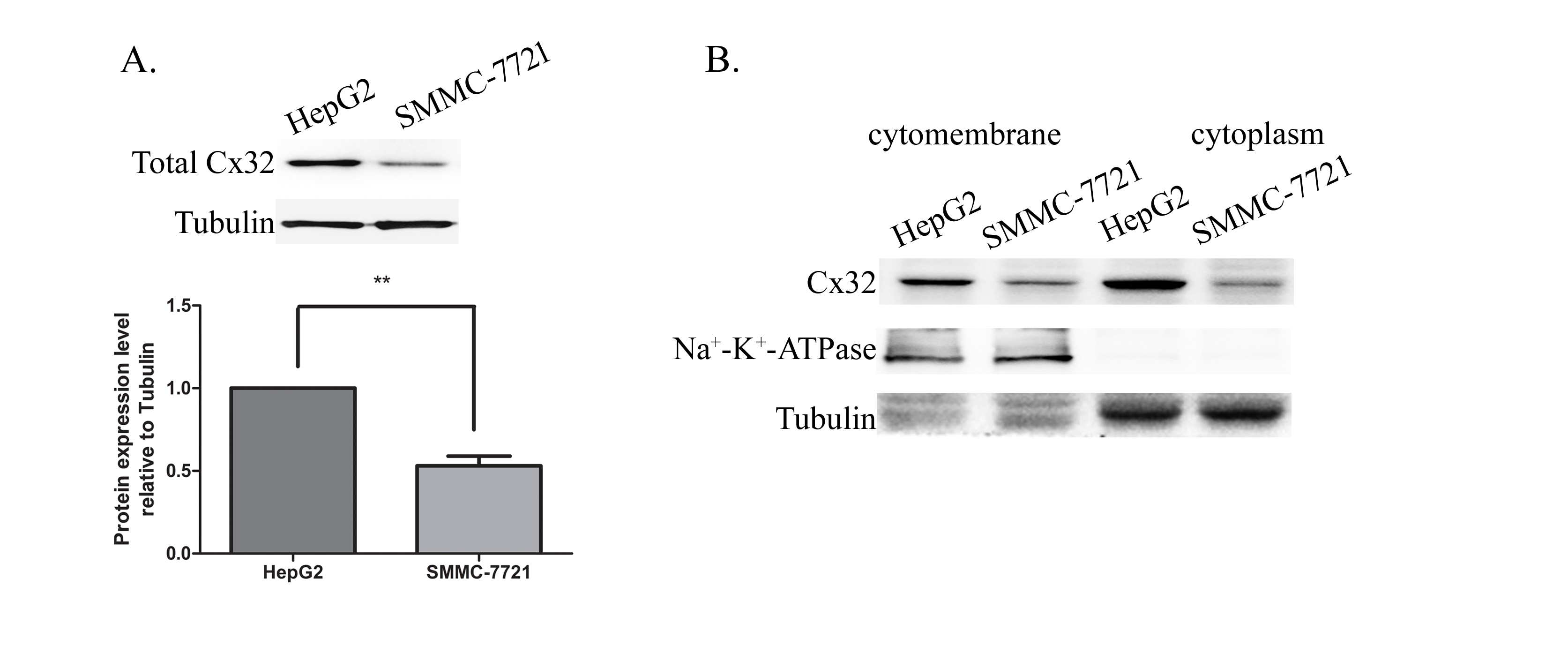

Supplement: Supplementary file 1 — Figure S1. The expression and distribution of Cx32 in HCC cell lines. Figure S2. The GJ function in HCC cell lines. Figure S3. The expression and distribution of Cx32 in HepG2-NC and HepG2-siCx32, SMMC-7721-vecotr and SMMC-7721-Cx32 cells. (ZIP 4027 kb) [file 13046_2019_1142_MOESM1_ESM.zip › Supplement Fig. S1.tif]

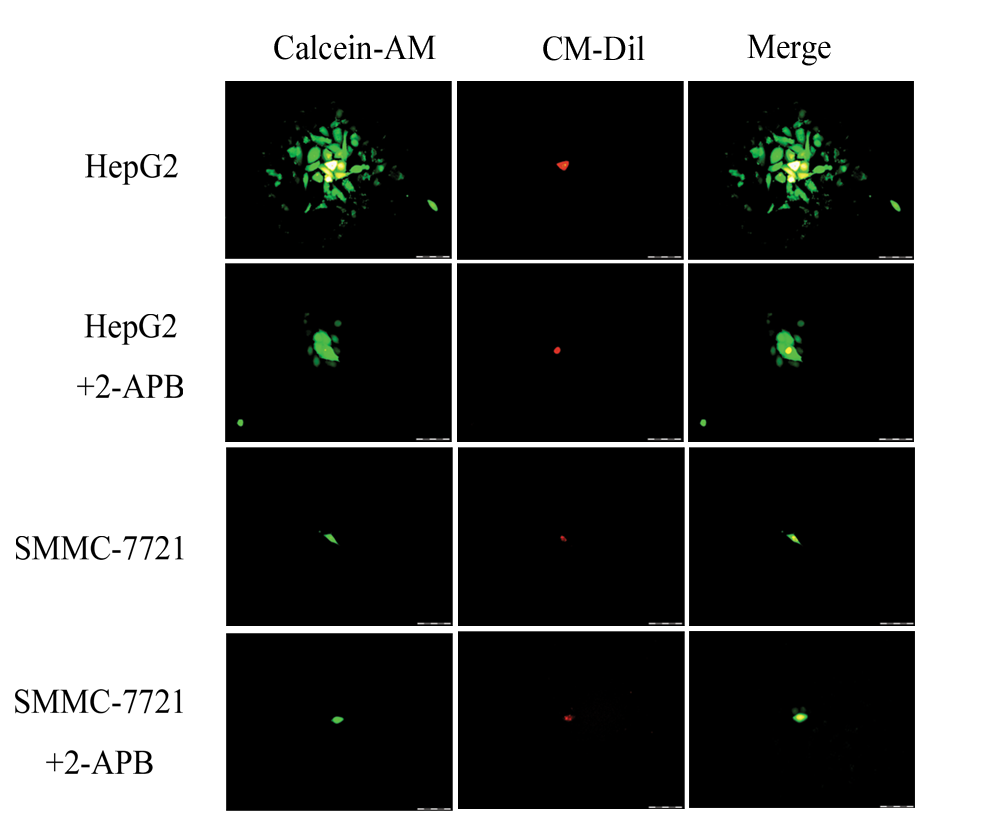

Supplement: Supplementary file 1 — Figure S1. The expression and distribution of Cx32 in HCC cell lines. Figure S2. The GJ function in HCC cell lines. Figure S3. The expression and distribution of Cx32 in HepG2-NC and HepG2-siCx32, SMMC-7721-vecotr and SMMC-7721-Cx32 cells. (ZIP 4027 kb) [file 13046_2019_1142_MOESM1_ESM.zip › Supplement Fig. S2.tif]

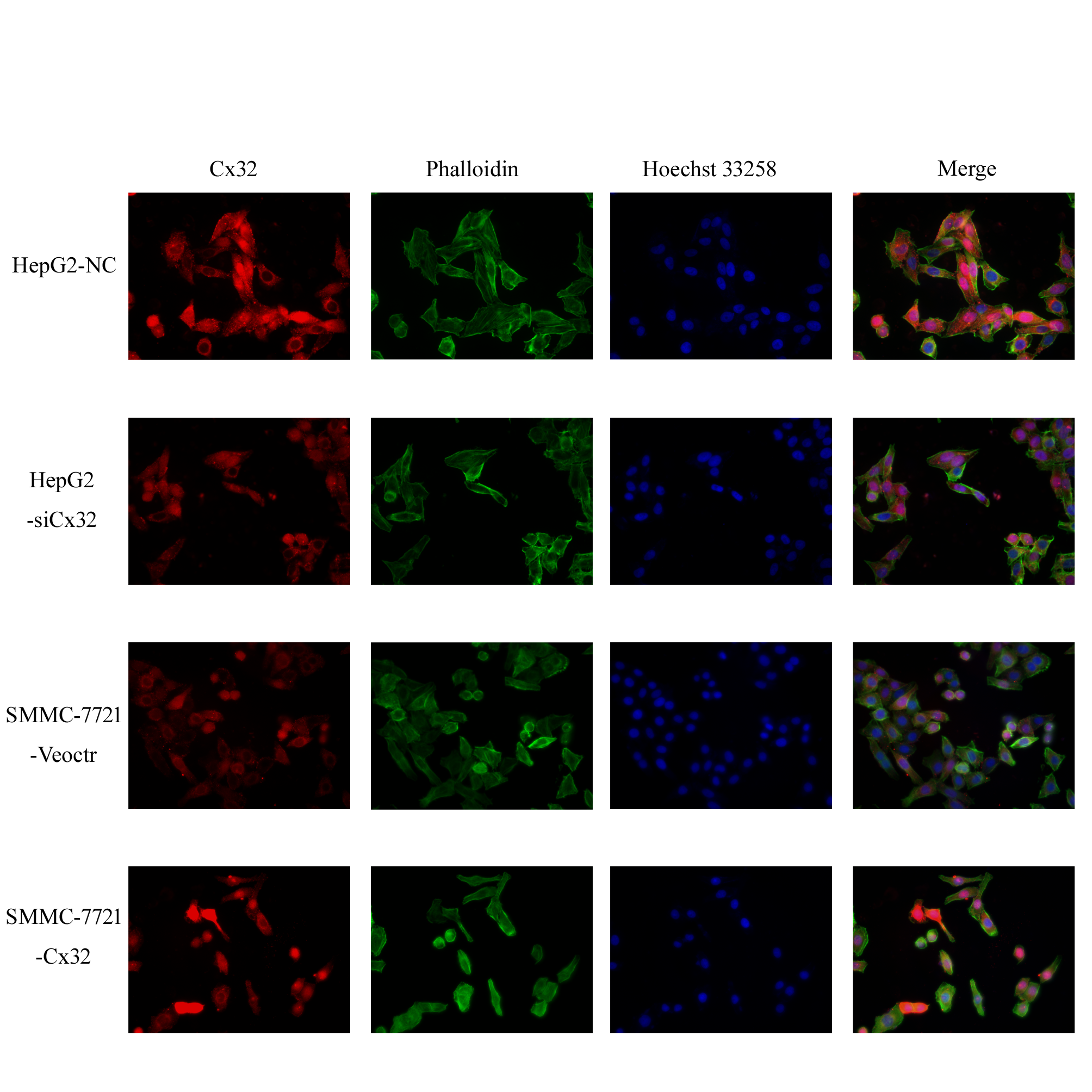

Supplement: Supplementary file 1 — Figure S1. The expression and distribution of Cx32 in HCC cell lines. Figure S2. The GJ function in HCC cell lines. Figure S3. The expression and distribution of Cx32 in HepG2-NC and HepG2-siCx32, SMMC-7721-vecotr and SMMC-7721-Cx32 cells. (ZIP 4027 kb) [file 13046_2019_1142_MOESM1_ESM.zip › Supplement Fig. S3.tif]
