# Supplementary material for: Cx32 exerts anti-apoptotic and pro-tumor effects via the epidermal growth factor receptor pathway in hepatocellular carcinoma
Source: J Exp Clin Cancer Res. 2019 Apr 4;38:145. doi: 10.1186/s13046-019-1142-y (PMC6449973; doi:10.1186/s13046-019-1142-y)
Supplement: Supplementary file 2 — Table S1. Cx32, Cx26 and Cx43 expression detected by immunohistochemical analysis in remote normal liver, peritumoral and HCC tissues. Table S2. Univariate Cox proportional hazard analysis for overall survival Variables. Table S3. Multivariate Analysis of Different Prognostic Parameters in Patients with HCC by Cox Regression Analysis. (DOCX 17 kb) [file 13046_2019_1142_MOESM2_ESM.docx]

**Supplementary Table S1: Cx32, Cx26 and Cx43 expression detected by IHC in remote normal liver, peritumoral and HCC tissues**

|  | **n** | **Low expression** | | **High expression** | | ***χ^2^*** | ***P*** |
| --- | --- | --- | --- | --- | --- | --- | --- |
|  |  | **0** | **+** | **++** | **+++** |  |  |
| **Cx32** |  |  |  |  |  |  |  |
| Normal | 42 | 2 | 25 | 11 | 4 | 0.040 | *P*1=0.842 |
| Peritumoral | 96 | 11 | 49 | 25 | 11 | 6.776 | *P*2=0.009** |
| HCC | 96 | 6 | 36 | 24 | 30 | 4.929 | *P*3=0.026* |
| **Cx26** |  |  |  |  |  |  |  |
| Normal | 42 | 4 | 11 | 14 | 13 | 0.007 | *P*1=0.933 |
| Peritumoral | 96 | 14 | 21 | 35 | 26 | 0.581 | *P*2=0.446 |
| HCC | 96 | 14 | 16 | 28 | 38 | 0.265 | *P*3=0.607 |
| **Cx43** |  |  |  |  |  |  |  |
| Normal | 42 | 2 | 16 | 12 | 12 | 0.017 | *P*1=0.896 |
| Peritumoral | 96 | 4 | 36 | 44 | 12 | 19.157 | *P*2=0.000** |
| HCC | 96 | 52 | 18 | 20 | 6 | 11.426 | *P*3=0.001** |

*P*1, Normal vs peritumoral; *P*2, peritumoral vs HCC; *P*3, normal vs HCC; *, *P*<0.05; **, *P*<0.01.

**Supplementary Table S2: Univariate Cox proportional hazard analysis for overall survival Variables**

| **Variables** | **Overall survival** | |
| --- | --- | --- |
|  | **RR (95% CI)** | ***P*** |
| Age, years (≤50 vs>50) | 1.371 (0.680 2.762) | 0.377 |
| Sex (Female vs Male) | 0.715 (0.313 1.632) | 0.425 |
| Differentiation (1/2 vs 3/4) | 0.450 (0.209 0.972) | 0.042* |
| TNM stage (Ⅰ/Ⅱ vs Ⅲ) | 0.664 (0.247 1.784) | 0.417 |
| Serum AFP, ng/ml (≤25 vs. >25) | 0.843 (0.395 1.799) | 0.658 |
| HBsAg (Negative vs Positive) | 0.295 (0.095 0.912) | 0.034* |
| Tumor size, cm (≤5 vs >5) | 0.498 (0.173 1.435) | 0.197 |
| Cx32 (low vs High) | 0.265 (0.120 0.586) | 0.001* |
| Cx43 (low vs High) | 1.161 (0.579 2.329) | 0.821 |
| Cx26 (low vs High) | 0.921 (0.451 1.881) | 0.673 |

RR, risk ratio; CI, confidence interval; * indicates *P*<0.05.

**Supplementary Table S3: Multivariate Analysis of Different Prognostic Parameters in Patients with HCC by Cox Regression Analysis**

| **Variables** | **B** | **SE** | **Wald** | **df** | **Sig.** | **RR(95%CI)** |
| --- | --- | --- | --- | --- | --- | --- |
| Differentiation (Ⅰ/Ⅱ vs Ⅲ/Ⅳ) | -0.945 | 0.356 | 7.057 | 1 | 0.008* | 0.389 (0.193 0.780) |
| Cx32 (low vs High) | -1.229 | 0.364 | 11.408 | 1 | 0.001* | 0.293(0.143 0.597) |

B, Regression coefficient; SE, standard error of regression coefficient; Wald, [chi-square](javascript:;) [value](javascript:;); df, degrees of freedom; Sig, P value; RR, risk ratio; CI, confidence interval; * indicate *P*<0.05.
