# Supplementary material for: Cx32 exerts anti-apoptotic and pro-tumor effects via the epidermal growth factor receptor pathway in hepatocellular carcinoma
Source: J Exp Clin Cancer Res. 2019 Apr 4;38:145. doi: 10.1186/s13046-019-1142-y (PMC6449973; doi:10.1186/s13046-019-1142-y)
Supplement: Supplementary file 3 — Supplemental Methods: immunofluorescence, cytomembrane protein extraction, parachute dye-coupling assay. (DOCX 14 kb) [file 13046_2019_1142_MOESM3_ESM.docx]

**Supplemental Methods**

**Immunofluorescence**

Cells were seeded on glass coverslips for 24 h and then fixed with 4% paraformaldehyde for 30 min and blocked with 2% BSA (USB Corporation, Cleveland, OH, USA) for 30 min. The cells were subsequently incubated with primary antibody directed against Cx32 (Santa Cruz, Dallas, TX, USA) (1:200) at 4 °C overnight, followed by incubation with Alex Flour 555-conjugated secondary antibody (Thermo Fisher, Waltham, MA, USA) (1:400) for 1 h in the dark. Alexa Fluor^®^ 488 Phalloidin (Cell Signaling, Danvers, MA, USA) (1:20) was applied for 15 min to stain the cytoskeleton. Hoechst 33258 (Sigma-Aldrich, St. Louis, MO, USA) (1 μg/ml) was used for nuclear staining. Then, the cells were washed with PBS and visualized under a fluorescence microscope (Olympus IX83, Tokyo, Japan).

**Cytomembrane protein extraction**

Cell membrane proteins and cytoplasmic proteins were isolated by using Mem-PER™ Plus Membrane Protein Extraction kit (ThermoFisher, Waltham, MA, USA) following the manufacturer's instructions.

**Parachute dye-coupling assay**

The assay for gap junction function was performed as described by Goldberg et al. HCC cells were seeded in a 12-well cell culture cluster and grown to 90% confluence. Then, the donor cells were double-labeled with 5 μM CM-DiI (Invitrogen, Carlsbad, CA, USA), a membrane dye that does not spread to coupled cells, and 5 μM calcein-AM (Invitrogen, Carlsbad, CA, USA), which is transferred between coupled cells through gap junctions. After culturing with CM-DiI and calcein-AM for 30 min at 37 °C, the donor cells were trypsinized and seeded onto receiver cells at a 1:150 donor/receiver ratio. The donor cells were allowed to attach to the monolayer of receiver cells and form GJ for 4 h at 37 °C and then examined with a fluorescence microscope (Olympus IX71, Tokyo, Japan). The average number of receiver cells containing calcein-AM per donor cell was regarded as a measure of the degree of GJ.
